# Effect of total flavonoids of *Spatholobus suberectus* Dunn on PCV2 induced oxidative stress in RAW264.7 cells

**DOI:** 10.1186/s12906-017-1764-6

**Published:** 2017-05-02

**Authors:** Hai-lan Chen, Jian Yang, Yuan-fang Fu, Xi-nan Meng, Wei-dan Zhao, Ting-jun Hu

**Affiliations:** Animal Science and Technological College, Guangxi University, Room 124, 100 Daxue Road, Nanning, Guangxi 530005 China

**Keywords:** Total flavonoids of *Spatholobus suberectus* Dunn, Porcine circovirus virus type 2, Oxidative stress, RAW264.7 cells, Antioxidant

## Abstract

**Background:**

This study was carried out to investigate the effect of total flavonoids of *Spatholobus suberectus* Dunn (TFSD) on PCV2 induced oxidative stress in RAW264.7 cells.

**Methods:**

Oxidative stress model was established in RAW264.7 cells by infecting with PCV2. Virus infected cells were then treated with various concentrations (25 mg/ml, 50 mg/ml and 100 mg/ml) of TFSD. The levels of oxidative stress related molecules (NO, ROS, GSH and GSSG) and activities of associated enzymes (SOD, MPO and XOD were analyzed using ultraviolet spectrophotometry, fluorescence method and commercialized detection kits.

**Results:**

PCV2 infection induced significant increase of NO secretion, ROS generation, GSSG content, activities of both XOD and MPO, and dramatically decrease of GSH content and SOD activity in RAW264.7 cells (*P* < 0.05). After treating with TFSD, PCV2 induced alteration of oxidative stress related molecule levels and enzyme activities were recovered to a level similar to control.

**Conclusion:**

Our findings indicated that TFSD was able to regulate oxidative stress induced by PCV2 infection in RAW264.7 cells, which supports the ethnomedicinal use of this herb as an alternative or complementary therapeutic drug for reactive oxygen-associated pathologies.

## Background

Increased generation of reactive oxygen species (ROS) and changes in redox homestasis have been reported in the context of many viral infections [1–6] and the failure to maintain an appropriate redox balance contributes to viral pathogenesis through alteration of biological structures and the massive induction of cell death [7]. Porcine circovirus type 2 (PCV2), a small, nonenveloped, single-stranded DNA virus, is the main pathogen of porcine circovirus diseases (PCVD) including porcine respiratory disease complex, enteric disease, reproductive disease, porcine dermatitis and nephropathy syndrome and postweaning multisystemic wasting syndrome (PMWS) [8–13]. In recent decades, PCVD caused huge economic losses on global swine industry. PCV2 infection induces oxidative stress and immunosuppression in pigs which further facilitate virus replication [14]. It has been reported that there was a time-dependent increase in ROS following PCV2 infection and oxidative stress induced by H_2_O_2_ enhanced PCV2 replication in PK-15 cells. Antioxidant N-acetyl-l-cysteine (NAC) treatment was able to inhibited PCV2 replication inside the kidney cells, whereas GSH depletion with buthionine sulfoximine (BSO) resulted in elevation of ROS levels and increased PCV2 replication [15]. PCV2 infection might be promoted by ROS-induced NF-κB activation, as inhibiting the activity of NF-κB, a redox-responsive transcription factor, suppressed BSO-mediated increase of PCV2 replication [15]. PCV2 infection induced elevation of ROS level and release of proinflammatory factors, such as IL-1β, IL-10, IL-8 and TNF-α, resulting in decrease of cell viability [16]. Previous studies in our laboratory showed that total superoxide dismutase (T-SOD) activity, total antioxidant capacity (TAOC) and GSH level of PCV2-infected mice spleen and thymus were significantly decreased [17]. Oxidative stress model induced by PCV2 has been successfully established in RAW264.7 cells which represented with remarkably elevation of NO level, MPO activity, iNOS expression and decrease of GSH/GSSG ratio, hydroxyl radical inhibitory capacity and cell viability [14].

The traditional method for viral diseases prevention is vaccination, which have disadvantages of limited protection period and cannot eradicate virus [18]. Besides, no vaccines are available for effective prevention of complicated disease such as PCVD. Thus, there is great demand for alternative methods to control viral disease. Since oxidative stress are often induced by virus infection, antioxidants are becoming promising candidate as therapeutic agents. For example, the thiol antioxidant of N-acetylcysteine amide (NACA) and antioxidant vitamins have been reported to effectively protect RBE4 cells or patients from HIV-1 induced toxicity by inhibiting oxidative stress formation [19, 20]. Previous studies found that the antoxidant trace element Selenium (Se) could affect the progression of some viral infections and suppress PCV2 replication in PK-15 cells [21–23]. Our previous studies found that carboxy methyl pachymaran (CMP) and *Sophora subprosrate* polysaccharide were able to regulate the immunity funtions and oxidative status by increasing the production of glutathione (GSH), superoxide dismutase (SOD) activity and total antioxidant capacity in PCV2 infected mice or RAW264.7 cells [14, 17].

*Spatholobus suberectus (S. suberectus)* Dunn is a widely used traditional medicines which possesses pharmacological activities of blood circulation improvement, antiplatelet, anti-inflammation, anti-bacterial, neuroprotection, and anti-cancer effects [24, 25]. Water extract component of *S. suberectus* Dunn showed strong free radical scavenging activity and antioxidative effect [26]. It is speculated that *S. suberectus* Dunn might be useful for the prevention and treatment of reactive oxygen-associated pathologies. There are many secondary compounds in *S. suberectus* Dunn, and flavonoids are the major bioactive substances, such as 3′,4′,7-trihydroxyglavone, formononetin, calycosin, prunetin, eriodictyol, butin, liquiritigenin, plathymenin, dihydroquercetin and dihydrokaempferol [27]. Effect of the flavonoids, the major bioactive substances of *S. suberectus* Dunn, on PCV2 induced oxidative stress both in vitro and in vivo has been not reported.

In the present study, the cellular toxicity of total flavonoids of *S. suberectus* Dunn (TFSD) was firstly evaluated and the regulatory role of TFSD on PCV2 induced oxidative stress in RAW264.7 cells was investigated.

## Methods

### Reagent

Vitamin C, dimethylsulfoxide (DMSO), sodium dodecyl sulfonate (SDS), naphthylethylenediamine dihydrochloride, sulphanilamide, phosphoric acid (H_3_PO_4_), ethylene diamine tetraacetic Acid (EDTA), 2′,7′-dichlorofluorescein diacetate (DCFH-DA), 3-4,5-dimethyl-2-thiazolyl)-2,5-diphenyl-2-H-tetrazoliumbromide (MTT), o-Phthalaldehyde (OPA), and n-ethylmaleimide (NEM) were obtained from Sigma, USA. High glucose DMEM medium, penicillin sodium and streptomycin were obtained from Gibco, USA. Fetal bovine serum (FBS) were purchased from PAN, Germany. Commercial kits for the analysis of superoxide dismutase (SOD), xanthine oxydase (XOD) and myeloperoxidase (MPO) were purchased from Nanjing Jiancheng Bioengineering Institute, China. All other reagents were analytical grade and used as received.

### TFSD preparation

*S. suberectus* Dunn which was collected in Chongzuo, Guangxi province, China in 2014, was purchased from the Chinese herbal medicine market in Zhongyao road in Nanning, Guangxi province. It was identified in the lab of pharmacology at Animal Science and Technology College, Guangxi University. The *S. suberectus* Dunn was first ground into coarse powder and total flavonoids of *S. suberectus* Dunn (TFSD) was then extracted from the coarse powder of *S. suberectus* Dunn using ethanol extraction. The total flavonoid content of obtained product has been determined to be 58.00% via ultraviolet spectrophotometry using rutin as standard as previously described [28, 29]. 5 mg of the yellow color TFSD was dissolved in PBS solution containing 1% of DMSO and filtered with a 0.22 μm membrane to prepare the stock solution (5 mg/mL). And then it was diluted to define concentration using complete medium upon used.

### Virus and cells

PCV2 was provided by the Key Laboratory of Animal Diseased Diagnostic and Immunology of Ministry of Agriculture at Nanjing Agricultural University and amplified using PK-15 cells. Titers of PCV2 were determined to be 10^4.7^ TCID_50_/0.1 mL using the Reed-Muench assay and diluted with the culture medium to 10^2.7^ TCID_50_ for the following experiments. PK-15 and RAW264.7 cells were purchased from the Type Culture Collection of Chinese Academy of Sciences, Shanghai, China. Both cells were cultured in DMEM supplemented with 10% of heat-inactivated FBS, 100 U/mL penicillin sodium and 100 μg/mL streptomycin in a humidified atmosphere at 37 °C, 5% CO_2_.

### Determination of the cellular toxicity of TFSD

The cytotoxicity of TFSD were analyzed using MTT assay. Briefly, RAW264.7 cells (1 × 10^5^ cells/well) were cultured in a 96-well plate overnight. The supernatant was removed and the cells were treated with various concentration of TFSD for 48 h. Supernatant was replaced with fresh medium containing 0.5 mg/mL MTT. After 4 h incubation at 37 °C, 100 μL 10% SDS solution with 0.01 M HCl was added to dissolve the purple crystals. After overnight incubation, the optical density (OD) at 595 nm were measured using a automatic microplate reader (PerkinElmer EnSpire). The viability of cells treated with TFSD was calculated as percentage of control.

### Establishment of PCV2 induced oxidative stress in RAW264.7 cells and TFSD treatment

RAW264.7 cells (1 × 10^6^ cells/well) were cultured in 24-well plates overnight. The supernatant was discard and the cell monolayer was washed with 0.1 M PBS (pH 7.2) for three times. The cells were then incubated with 10^2.7^ TCID_50_ PCV2 for 2 h to allow virus adhere to and enter cells. The virus was removed and cells were washed with PBS for three times, followed by treating with TFSD in concentrations of 25, 50 or 100 μg/mL. Completed DMEM medium without TFSD was added into control group without PCV2 infection and model group with PCV2 infection. Vitamin C (Vc) was used as the drug for positive control. The cells were further cultured for 12 h.

### Analysis of nitric oxide (NO) secretion

The secretion of NO was studied by a spectrophotometric assay based on the Griess reaction [30]. Briefly, 100 μl of the culture supernatant was mixed with an equal volume of Griess reagent (freshly mixed 0.1% naphthylethylenediamine dihydrochloride solution and 1% sulphanilamide in 5% H_3_PO_4_ solution in a volume ratio of 1:1) at room temperature. The mixture was allowed to react for 15 min at room temperature, and the absorbance at 540 nm was measured on an automatic microplate reader (PerkinElmer EnSpire). The NO concentration was determined by a standard curve of NaNO_2_.

### Fluorescence assay of ROS

The cells were grown in black 96-well plates with transparent bottom for the analysis of ROS using the fluorescent probe of DCFH-DA [31]. After removing the culture medium, the cells were washed with PBS for three times. 50 μL of DCFH-DA (10 μM/L) was added into each well and incubated in dark for 30 min at 37 °C. The Cells were washed with PBS for three times and fluorescent intensity was measured at 485 nm for excitation and 530 nm for emission on an automatic microplate reader (PerkinElmer EnSpire).

### Detection of intracellular GSH and oxidized glutathione (GSSG)

The cells were scraped down from the bottom of 24-well plates and collected by centrifuging at 2000 rpm for 5 min. The cell pellet was resuspended with 0.4 mL of 5% trichloroacetic acid (TCA) and ultrasonic decomposed in an ice-water bath for 1.0 min with 2 s on and 2 s off. The cell lysate was centrifuged at 12,000 rpm for 15 min at 4 °C and the supernatant was used for GSH and GSSG assays. 3.6 mL phosphate-EDTA buffer (pH 8.0) and 200 μL OPA (1 mg/mL) was added into 200 μL of the supernatant and incubated for 40 min at room temperature. Fluorescent intensity was measured using a automatic microplate reader (PerkinElmer EnSpire) at an excitation wavelength of 350 nm and an emission wavelength of 425 nm. For the GSSG analysis, 40 μL NEM (0.04 mol/L) was added to another 100 μL of supernatant and incubated for 30 min at room temperature. 1.9 ml of NaOH (0.1 mol/L) and 100 μL of OPA (1 mg/mL) were added into the mixture and incubated for another 15 min at room temperature. Fluorescent intensity were measured with excitation wavelength of 337.8 nm and emission wavelength of 421.6 nm on an automatic microplate reader (PerkinElmer EnSpire). The concentrations of both GSH and GSSG were determined by standard curves of GSH and GSSG.

### Determination of activities of SOD, XOD and MPO

The activities of intracellular superoxide dismutase (SOD), xanthine oxydase (XOD) and myeloperoxidase (MPO) were evaluated using commercial kits following the manufacturer’s instructions.

### Statistical analysis

Statistical analysis was performed using the software of SPSS version 17.0. Data were analyzed using one-way analysis of variance (ANOVA) followed by the Duncan test. Data are expressed as means ± SD. Differences were regarded as significant at *P* < 0.05.

## Results

### Cytotoxicity of TFSD on RAW264.7 cells

**Fig. 1:**
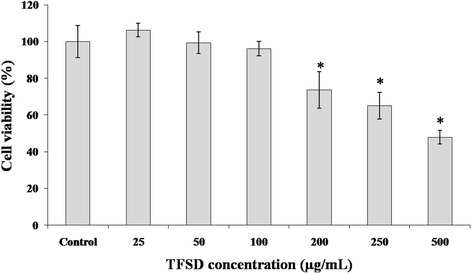
Viability of RAW264.7 cells after treating with various concentrations of TFSD for 48 h. Data are presented as mean ± S.D. *Bars* with * indicate statistically different from control (*P* < 0.05)

Before further studies were conducted, TFSD was firstly tested for its effect on the cellular viability of RAW264.7 cells using MTT assay (Fig. 1). When the concentration of TFSD was lower than 100 μg/mL, the cell viability were greater than 80% and no significant difference has been observed when compared to control. However, as the concentration of TFSD increased to a level higher than 200 μg/mL, the viability of RAW264.7 cells were significantly decreased, indicating inhibition of cell proliferation by TFSD. Thus, concentrations of 25, 50 and 100 μg/mL were selected for further study.

### NO contents and ROS production

**Fig. 2:**
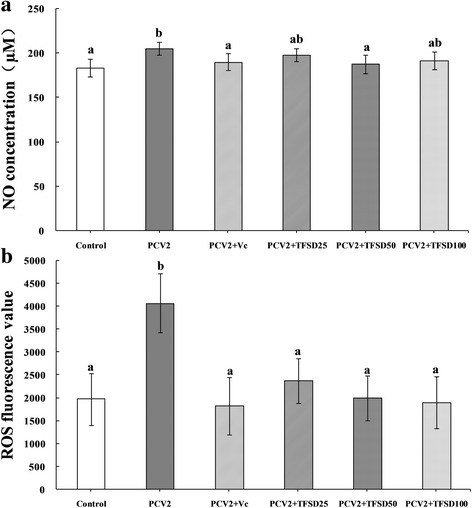
Effect of TFSD on NO secretion (**a**) and ROS production (**b**) in PCV2 infected RAW264.7 cells. Data are presented as mean ± S.D. *Bars* with different *letters* are statistically different (*P* < 0.05). Control: cells without PCV2 infection and drug treatment; PCV2: cells infected with 10^2.7^ TCID_50_ PCV2; PCV2 + Vc: cells treated with 100 μg/mL of vitamin C after PCV2 infection; PCV2 + TFSD25–100: cells treated with TFSD at concentrations of 25, 50 or 100 μg/mL after PCV2 infection, respectively

Regulatory effects of TFSD on NO contents and ROS production in PCV2 infected cells were showed in Fig. 2. Compared to control, PCV2 infection induced significantly up-regulated NO secretion and intracellular ROS production. Vitamin C, a widely used antioxidant, was able to inhibit the elevation of NO and ROS content induced by PCV2 infection. Cells treated with TFSD exhibited similar results to those treated with Vc. Compared to PCV2 group, NO secretion in 50 μg/mL TFSD treatment group and ROS production in all TFSD treated groups was significantly decreased (*P* < 0.05) (Fig. 2).

### GSH and GSSG contents

**Fig. 3:**
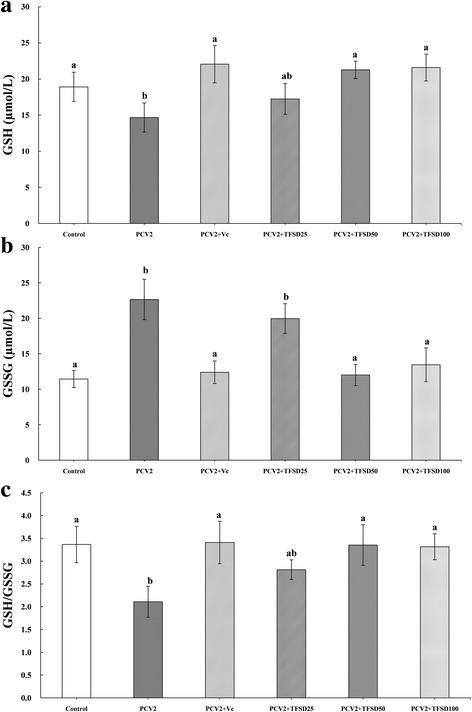
Effect of TFSD on intracellular GSH concentration (**a**), GSSG content (**b**) and GSH/GSSG ratio (**c**) in PCV2 infected RAW264.7 cells. Data are presented as mean ± S.D. *Bars* with different *letters* are statistically different (*P* < 0.05). Control: cells without PCV2 infection and drug treatment; PCV2: cells infected with 10^2.7^ TCID_50_ PCV2; PCV2 + Vc: cells treated with 100 μg/mL of vitamin C after PCV2 infection; PCV2 + TFSD25–100: cells treated with TFSD at concentrations of 25, 50 or 100 μg/mL after PCV2 infection, respectively

Effects of TFSD on intracellular GSH and GSSG content are shown in Fig. 3. The intracellular GSH level was significantly decreased after infected with PCV2 (Fig. 3a) and treatment with TFSD at concentrations of 50 and 100 μg/mL was able to inhibit the reduction of GSH level (*P* < 0.05). In contrast, PCV2 infection resulted in an elevation of intracellular GSSG content, while TFSD treatment (50 and 100 μg/mL) was able to decrease GSSG level compared to PCV2 group (Fig. 3b). In addition, the ratio of GSH to GSSG showed similar trend to that of GSH content, which was significantly increased in virus infected cells but recovered to a level close to control after TFSD treatment (Fig. 3c).

### SOD activity

**Fig. 4:**
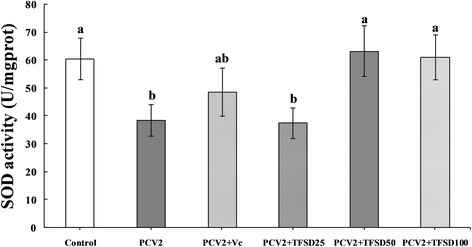
Effect of TFSD on SOD activity in PCV2 infected RAW264.7 cells. Data are presented as mean ± S.D. *Bars* with different *letters* are statistically different (*P* < 0.05). Control: cells without PCV2 infection and drug treatment; PCV2: cells infected with 10^2.7^ TCID_50_ PCV2; PCV2 + Vc: cells treated with 100 μg/mL of vitamin C after PCV2 infection; PCV2 + TFSD25–100: cells treated with TFSD at concentrations of 25, 50 or 100 μg/mL after PCV2 infection, respectively

Compared to control, the SOD activity in RAW264.7 cells was significantly decreased when cells were infected with PCV2 (*P* < 0.05) (Fig. 4). Antioxidant (Vc and TFSD) treatment was able to inhibit the reduction of SOD activity. In 50 and 100 μg/mL TFSD groups, the SOD activity was significantly higher than that of PCV2 group (*P* < 0.05).

### XOD and MPO activities

**Fig. 5:**
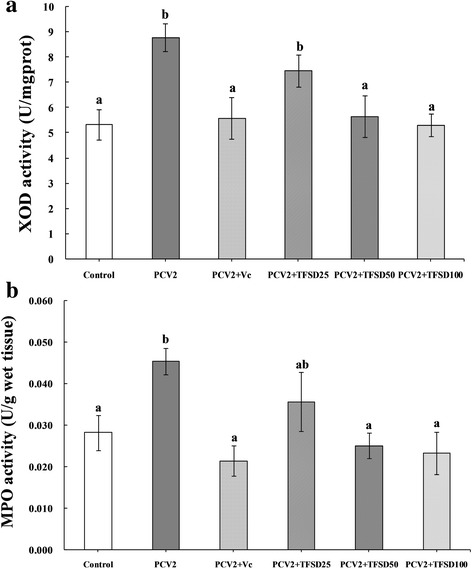
Effect of TFSD on activities of XOD (**a**) and MPO (**b**) in PCV2 infected RAW264.7 cells. Data are presented as mean ± S.D. *Bars* with different *letters* are statistically different (*P* < 0.05). Control: cells without PCV2 infection and drug treatment; PCV2: cells infected with 10^2.7^ TCID_50_ PCV2; PCV2 + Vc: cells treated with 100 μg/mL of vitamin C after PCV2 infection; PCV2 + TFSD25–100: cells treated with TFSD at concentrations of 25, 50 or 100 μg/mL after PCV2 infection, respectively

PCV2 infection caused significantly increase of XOD and MPO activities (Fig. 5) while treatment of infected cells with TFSD at concentrations of 50 and 100 μg/mL was able to promoted the activity of XOD and MPO. The recovery of XOD and MPO activities to a level similar to control by treating with antioxidant (Vc and TFSD) suggested that antioxidant can protect RAW264.7 cells from oxidative stress damage induced by PCV2 infection.

## Discussions

Upon viral infections, the antiviral and inflammatory signaling pathways will be activated, which has been reported to associate with the production of ROS [1, 32–35]. For example, infection of Kaposi’s sarcoma-associated herpesvirus (KSHV) or H5N1 induced ROS production and signaling pathways amplification, which facilitated virus invasion and replication [36, 37]. Oxidative stress with increased inflammatory cytokines secretion was reported in Dengue virus infected patients [38], while the oxidative stress induced damage and alterations in redox status are believed to related to increasing severity of the disease [39, 40]. PCV2, the main pathogen accounts for PMWS, has been reported to infected PK-15 cells, RAW264.7 cells and mice to induce oxidative stress [14, 17, 22]. In the present study, the levels of intracelluar oxidants (NO, ROS, GSSG) and activities of intracellular oxidase (XOD and MPO) were significantly increased, while the antioxidant species content (GSH) and antioxidase activity (SOD) was remarkably decreased in RAW264.7 cells after infected with PCV2 at a titer of 10^2.7^ TCID_50_, indicating the creation of oxidative stress in infected cells.

In macrophages, NO is synthesised from L-arginine and excess NO interact with oxygen radicals to produce peroxynitrite (ONOO^−^) which might lead to proteins injury, DNA damage and phospholipid membranes damage [41, 42]. Increased production of NO has been observed during viral infection, which in turn promotes viral replication [43, 44]. On the other hand, accumulated ROS not only helps to viral invasion and/or replication but also leads to cell injury by attacking biomacromolecules which can lead to immunosuppression and cell apoptosis [45]. The damage of biomacromolecules generates extra ROS which in turn aggravates oxidative stress status [46, 47]. In addition, the effects of NO and ROS can be synergistic by depleting the intracellular antioxidant glutathione. It was assumed that PCV2 infection induced accumulation of NO and ROS accounts for the decrease in cell viability [14]. Thus, clearance of excess NO and ROS and preventing their accumulation is highly important. In this study, PCV2 infection induce significantly elevated production of NO and accumulation of intracellular ROS (Fig. 2), agreed with previous studies. TFSD treatment was able to inhibit the PCV2 induced increase of NO and ROS, suggesting that TFSD might protect cells from damage caused by excess NO and ROS.

Normally, ROS can be cleared away by oxidizing intracelluar GSH to GSSG, and thus GSH to GSSG ratio is an important indicator of cell antioxidant capacity [48]. Decreased extracellular and intracellular ratio of GSH to GSSG has been observed in patients infected by HCV or Dengue virus, suggesting enhanced glutathione turnover in the liver, blood and lymphatic system of infected patients [38, 49, 50]. Figure 3 shows that TFSD treatment was able to increase GSH level and the ratio of GSH to GSSG in PCV2 infected RAW264.7 cells, suggesting that TFSD has antioxidant effect which can recover the intracellular oxidative status, agreed with Matthaiou’s report that *Pomegranate* juice, which contains majorly flavonoids, can increase GSH levels in human blood [51].

Antioxidant enzymes are the primary defense that prevents biological macromolecules from oxidative stress induced damage. SOD has been considered as one of the most important enzymes in the enzymatic antioxidant defense system. It catalyzes the dismutation of superoxide radicals to produce H_2_O_2_ and molecular oxygen, and thus protects against oxidative processes initiated by the superoxide anion. Generation of free radicals such as superoxides is believed to play an essential role in the pathogenesis of various infectious diseases [52]. Decreased SOD activities have been detected in PCV2 infected splenic lymphocytes and RAW264.7 cells in vitro [14, 53]. In the current study, SOD activity was significantly decreased in PCV2 infected RAW264.7 cells, while such enzyme activity reduction can be reversed by treatment with 50 or 100 μg/mL TFSD, suggesting that TFSD can clear away ROS and increase activities of antioxidase in PCV2 infected disease.

MPO, a member of the super family of mammalian heme peroxidase enzymes, catalyze the H_2_O_2_-mediated oxidation of halide ions to hypohalous acids (HOCl) to kill invaded microbials. However, high concentration of HOCl will oxidize biomacromolecules and thus resulted in damage to normal tissues. Higher plasma MPO was found upon hepatitis B virus (HBV) infection which was thought to account for the liver injury in infected patients [14]. XOD is the enzyme responsible for the metabolism of hypoxanthine and xanthine to uric acid. Superoxide radicals were produced during this metabolism which will attack biomacromolecules to induce damage and extra ROS. In this study, MPO and XOD activities were significantly increased upon PCV2 infection which might contributed to the elevation of ROS content and decreased of GSH level. Treating with TFSD was able to inhibit the virus induced alteration of MPO and XOD activities, suggesting that TFSD can protect RAW264.7 cells from damages caused by PCV2 infection. These results are consistent with the report that flavonoids was able to inhibit XOD activity and clear away superoxide [54–56].

## Conclusions

In conclusion, oxidative stress was established in PCV2 infected RAW264.7 cells, represented by significantly increased NO secretion, intracellular ROS content, MPO activity, XOD activity, and remarkably reduced GSH levels, GSH/GSSG ratio, SOD activity. Treatment of infected cells with TFSD dramatically increased GSH level, GSH to GSSG ratio and SOD activity, reduced the intracellular ROS content and inhibited the MPO and XOD activity. All these results suggest that TFSD is an antioxidant candidate for the prevention and treatment of oxidative stress associated disease, including disease caused by virus infection.

## Abbreviations

CMP: Carboxy methyl pachymaran
DCFH-DA: 2′,7′-dichlorofluorescein diacetate
DMSO: Dimethylsulfoxide
EDTA: Ethylene diamine tetraacetic acid
FBS: Fetal bovine serum
GSH: Glutathione
GSSG: Oxidized glutathione
HOCl: Hypohalous acids
KSHV: Kaposi’s sarcoma-associated herpesvirus
MPO: Myeloperoxidase
MTT: 3-4,5-dimethyl-2-thiazolyl)-2,5-diphenyl- 2-H-tetrazoliumbromide
NACA: N-acetylcysteine amide
NEM: n-ethylmaleimide
NO: Nitric oxide
OD: Optical density (OD)
ONOO^−^: Peroxynitrite
OPA: o-Phthalaldehyde (OPA)
PCV2: Porcine circovirus virus type 2
PCVD: Porcine circovirus diseases
PK-15: Porcine kidney cells
PMWS: Postweaning multisystemic wasting syndrome
ROS: Reactive oxygen species
*S. suberectus*: *Spatholobus suberectus*
SDS: Sodium dodecyl sulfonate
Se: Selenium
SOD: Superoxide dismutase
TAOC: Total antioxidant capacity
TCA: Trichloroacetic acid
TFSD: Total flavonoids of *Spatholobus suberectus* Dunn
T-SOD: Total superoxide dismutase
XOD: Xanthine oxydase
